# Full-length transcriptome sequencing of pepper fruit during development and construction of a transcript variation database

**DOI:** 10.1093/hr/uhae198

**Published:** 2024-07-24

**Authors:** Zhoubin Liu, Bozhi Yang, Tianyuan Zhang, Hao Sun, Lianzhen Mao, Sha Yang, Xiongze Dai, Huan Suo, Zhuqing Zhang, Wenchao Chen, Hu Chen, Wangjie Xu, Komivi Dossa, Xuexiao Zou, Lijun Ou

**Affiliations:** Engineering Research Center of Education, Ministry for Germplasm Innovation and Breeding New Varieties of Horticultural Crops, Key Laboratory for Vegetable Biology of Hunan Province, College of Horticulture, Hunan Agricultural University, Changsha 410125, China; Engineering Research Center of Education, Ministry for Germplasm Innovation and Breeding New Varieties of Horticultural Crops, Key Laboratory for Vegetable Biology of Hunan Province, College of Horticulture, Hunan Agricultural University, Changsha 410125, China; Vegetable Institution of Hunan Academy of Agricultural Science, Changsha 410125, China; Wuhan Benagen Technology Co., Ltd., Wuhan 430075, China; Engineering Research Center of Education, Ministry for Germplasm Innovation and Breeding New Varieties of Horticultural Crops, Key Laboratory for Vegetable Biology of Hunan Province, College of Horticulture, Hunan Agricultural University, Changsha 410125, China; Engineering Research Center of Education, Ministry for Germplasm Innovation and Breeding New Varieties of Horticultural Crops, Key Laboratory for Vegetable Biology of Hunan Province, College of Horticulture, Hunan Agricultural University, Changsha 410125, China; Engineering Research Center of Education, Ministry for Germplasm Innovation and Breeding New Varieties of Horticultural Crops, Key Laboratory for Vegetable Biology of Hunan Province, College of Horticulture, Hunan Agricultural University, Changsha 410125, China; Engineering Research Center of Education, Ministry for Germplasm Innovation and Breeding New Varieties of Horticultural Crops, Key Laboratory for Vegetable Biology of Hunan Province, College of Horticulture, Hunan Agricultural University, Changsha 410125, China; Engineering Research Center of Education, Ministry for Germplasm Innovation and Breeding New Varieties of Horticultural Crops, Key Laboratory for Vegetable Biology of Hunan Province, College of Horticulture, Hunan Agricultural University, Changsha 410125, China; Vegetable Institution of Hunan Academy of Agricultural Science, Changsha 410125, China; Vegetable Institution of Hunan Academy of Agricultural Science, Changsha 410125, China; Wuhan Benagen Technology Co., Ltd., Wuhan 430075, China; Wuhan Benagen Technology Co., Ltd., Wuhan 430075, China; CIRAD, UMR AGAP Institut, 97170 Petit Bourg, Guadeloupe, France; Engineering Research Center of Education, Ministry for Germplasm Innovation and Breeding New Varieties of Horticultural Crops, Key Laboratory for Vegetable Biology of Hunan Province, College of Horticulture, Hunan Agricultural University, Changsha 410125, China; Engineering Research Center of Education, Ministry for Germplasm Innovation and Breeding New Varieties of Horticultural Crops, Key Laboratory for Vegetable Biology of Hunan Province, College of Horticulture, Hunan Agricultural University, Changsha 410125, China

## Abstract

Chili pepper is an important spice and a model plant for fruit development studies. Large-scale omics information on chili pepper plant development continues to be gathered for understanding development as well as capsaicin biosynthesis. In this study, a full-spectrum transcriptome data of eight chili pepper tissues at five growth stages using the Oxford Nanopore long-read sequencing approach was generated. Of the 485 351 transcripts, 35 336 were recorded as reference transcripts (genes), while 450 015 were novel including coding, lnc, and other non-coding RNAs. These novel transcripts belonged to unknown/intergenic (347703), those retained introns (26336), and had multi-exons with at least one junction match (20333). In terms of alternative splicing, retained intron had the highest proportion (14795). The number of tissue-specific expressed transcripts ranged from 22 925 (stem) to 40 289 (flower). The expression changes during fruit and placenta development are discussed in detail. Integration of gene expression and capsaicin content quantification throughout the placental development clarifies that capsaicin biosynthesis in pepper is mainly derived from valine, leucin, and isoleucine degradation as well as citrate cycle and/or pyrimidine metabolism pathways. Most importantly, a user-friendly Pepper Full-Length Transcriptome Variation Database (PFTVD 1.0) (http://pepper-database.cn/) has been developed. PFTVD 1.0 provides transcriptomics and genomics information and allows users to analyse the data using various tools implemented. This work highlights the potential of long-read sequencing to discover novel genes and transcripts and their diversity in plant developmental biology.

## Introduction

Chili pepper (*Capsicum annuum* L.) is the most widely grown spice and vegetable crop in the world. The area under cultivation in China exceeds 2 million hectares and produces more than 30% of the world’s chilies (www.fao.org). Chili peppers are a rich source of antioxidants (phenolics, flavonoids, etc.), natural colorings, vitamins, minerals and especially capsaicinoids [1]. In addition to its importance as a food source, research on chili pepper has contributed greatly to biological studies on evolution, biosynthesis of secondary metabolites, pigments and fruit development biology [2]. Its genome was assembled and 34 903 protein-coding genes were reported [3], providing a genome-wide view and understanding of both the *C. annuum* and *Capsicum chinense* genomes. Second generation sequencing techniques such as the Illumina, SOLiD, Ion Torrent, and 454 pyrosequencing, have further improved the quality of the reference genomes and provided detailed understanding of the evolution of *Capsicum* species [4]. To facilitate biological studies on chili pepper, omics databases are a useful resource for scientists. For example, the Sol Genomics Network (www.solgenomics.net) hosts the genome sequences of UCD-10X-F1 (a wide cross between Cariollos de Morelos 334 and a non-pungent blocky pepper breeding line) [5], the Mexican *C. annuum* landrace CM334 [3], the Mexican wild pepper accession ‘Chiltepin’, and the Chinese inbred derivative ‘Zunla-1’ [6]. However, these datasets mainly focus on the genome sequence and variations with little or no information on transcript variation. Although some databases present data for transcriptome and expression such as ‘The Capsicum Transcriptome DB’ which hosts a collection of expressed sequence tags of five chili pepper organs [7]. To further advance the availability of expression data, our team had previously established an informatics hub, ‘PepperHub’, which hosts the genome, transcriptome, sRNome, Variome, and Proteome [8]. Collectively, these large databases have increased the availability of pepper omics data. The ‘PepperHub’ offers transcriptome sequence originating from Illumina short-reads but the structural variations’ data is almost non-existent for chili peppers.

Capsaicin, found in chili peppers, is responsible for their heat and irritation. Over the last two decades, and since the widespread use of omics technologies, our understanding of the biosynthesis of capsaicin has improved significantly [3]. Capsaicin is condensed from vanillylamine, which is derived from phenylalanine. It accumulates in the developing fruit in the epidermal cells of the placenta and in blisters on the surface of the placenta [9]. A series of enzymatic reactions in the branched-chain fatty acid synthesis pathway produces 8-methyl-6-nonenoyl-CoA, which is converted to capsaicin [10]. However, the relationship between its accumulation in developing fruits and the expression patterns of genes involved in pathways converging on capsaicin biosynthesis is still unclear. Some studies have proposed that capsaicin accumulation reaches its maximum concentration at 30 days post-anthesis, while in adult plants it is 40 days post-anthesis [11]. However, a detailed understanding of capsaicin accumulation in the placenta of developing fruits and expression changes in the transcriptome is still lacking. RNA sequencing is a widely used quantitative approach to explore global gene expression changes that may contribute during fruit development [2]. RNA sequencing can also reflect tissue specificity, allowing exploration of highly regulated genes associated with characteristics of individual tissues [12]. Compared to second-generation transcriptomics [8], the use of long-read sequencing can be a better choice for dissecting complexity of pathways and RNA structures [13].

In this study, a systematic transcriptomic analysis of eight chili pepper tissues/organs at five plant growth stages was performed by employing Oxford Nanopore Technologies (ONT). Furthermore, the results were complemented with high-performance liquid chromatography and quantitative real-time PCR. Based on these techniques, we provide a comprehensive resource of full-spectrum chili pepper transcripts. Using this dataset, the expression changes during fruit and placenta development were systematically investigated. Moreover, we discussed how expression changes in pathways converging on capsaicin biosynthesis correlate with its content in the placenta. To provide free access to the full spectrum of transcriptome data, a user-friendly database, i.e. the ‘Pepper Transcriptome Variation Database’ (PFTVD 1.0) has been developed and implemented. PFTVD 1.0 seamlessly provides functions for searching, visualizing, browsing and downloading chili pepper transcriptome data through a simple, easy-to-use, intuitive and interactive graphical web interface.

## Results

### Full-spectrum transcriptome of chili pepper

**Figure 1:**
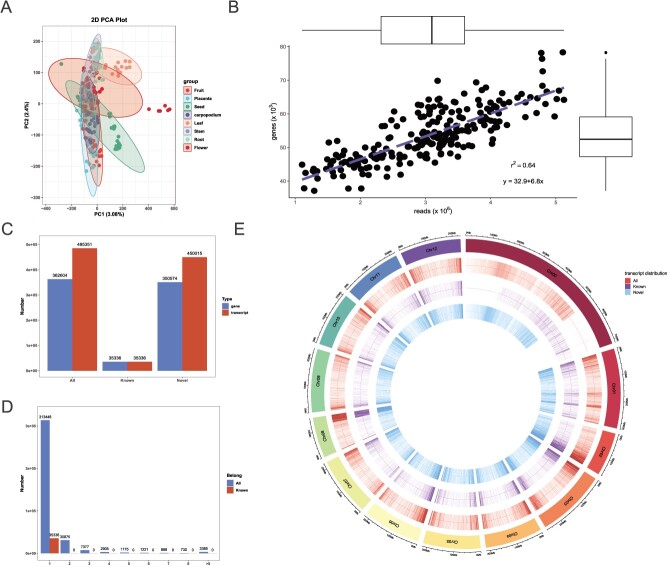
Preliminary analysis of full-spectrum chili pepper transcriptome. **A** Principal component analysis of TPM of genes. **B** Pearson’s correlation between the number of sequenced reads and the number of expressed genes. **C** Bar plot showing number of genes and transcripts. **D** Bar plot showing number of genes containing different number of transcripts. **E** Transcript density distribution map.

Total RNA was extracted from triplicate 76 biological samples of stem (S), leaf (L), root (R), fruit (FA), placenta (FB), seed (FC), carpopodium (FD), and flower (FL) collected in five stages: seed stage, seedling stage, flowering period, bud stage, and fruiting period. ONT full-length sequencing of the 228 cDNA libraries yielded 1658.27 million reads. The mean read length, N50, and meanQ were 1080.13 bp, 1362, and 11, respectively. Of these, 1559.45 million reads were filtered as clean reads, and further processing resulted in 1259.941 million full length reads, which were then aligned to the reference genome. On an average, 88.74% reads could be mapped to the reference genome (Table S1). Principal component analysis (PCA) together with hierarchical clustering of normalized gene expression showed that the samples were clustered by tissue (Fig. 1A). An exception was some FL samples—FL2-C (stamen at bud stage) and FL3-C (stamen at flowering period)—which did not cluster with the rest of the tissues/samples. There was a positive correlation (r = 0.64) between the number of sequenced reads and the number of expressed genes (Fig. 1B).

In general, 485 351 transcripts were identified. Of these, 35 336 were recorded as reference transcripts (genes) and 450 015 were novel (Fig. 1C). The genes contained a variable number of transcripts (1 to ≥8) (Fig. 1D). The novel transcripts were distributed on all 12 chromosomes such that chr. 1 had the maximum number of novel transcripts (32 106) and chr. 8 had minimum (17 162) (Fig. 1E). The transcripts were of three types: coding RNA (127 882), lncRNA (236 722), and other non-coding RNAs (120 747). The largest number of coding RNAs were protein-coding (101 151), followed by processed transcripts (16 250), non-stop decay (5321), and others. Of the long non-coding transcripts, 89.45% (205 632), 4.6% (10 572), 3.94% (9049), and 2.01% (4623) were lincRNAs, intronic-lncRNAs, antisense-lncRNAs, and sense-lncRNAs, respectively (Fig. S1, see online supplementary material).

**Figure 2:**
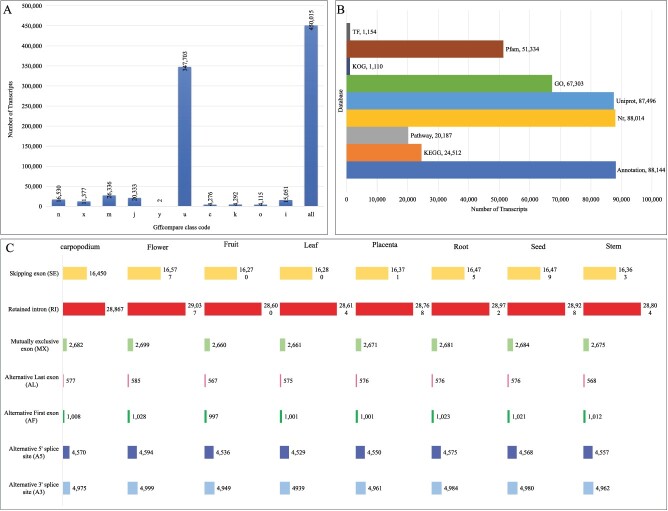
Number of transcripts as a function of Gffcompare codes and alternative splicing events in chili pepper. **A** GffCompare based classification of the novel transcripts. **B** Functional annotation statistic of novel transcripts. **C** Alternative splicing type and events in each tissue.

GffCompare classified the novel transcripts into different classes (Fig. 2A); of these, 347 703 were unknown/intergenic, while the maximum number of novel transcripts (26 336) retained intron(s). The third highest number of novel transcripts (20 333) had multi-exons with at least one junction match. Next, TransDecoder predicted 82 733 non-redundant CDS of new transcripts from 45 168 genes. By including known and novel transcripts, a total of 115 268 non-redundant CDSs were predicted from 77 923 genes. Of these, 92 546 novel transcripts, 94.86% had at least one annotated transcript (Fig. 2B; Fig. S2, see online supplementary material). In terms of alternative splicing (AS), seven types of AS events including alternative 3′ splice site (A3), alternative 5′ splice site (A5), alternative first exon (AF), alternative last exon (AL), mutually exclusive exon (MX), retained intron (RI), and skipped exon (SE) were found (Fig. S3A, see online supplementary material). The RI had the highest proportion (14 795) of AS events (Fig. S3B, see online supplementary material). The number of each type of AS events in the analysed chili pepper tissues is shown in Fig. 2C.

### Tissue-specific expression and differential alternative splicing

A transcript was considered tissue-specific if transcripts with TPM >1 were present in any five samples in that particular tissue [14]. The number of tissue-specific expressed transcripts ranged from 22 925 (stem) to 40 289 (flower) (Fig. S4Ai, see online supplementary material). Differences in average TPM between tissues were also noted (Fig. S4Aii, see online supplementary material). For instance, a novel transcript (*novel261770.t1*, KAF3652576.1 hypothetical protein FXO37_17464) was identified (Fig. S4Aiii, see online supplementary material). It was expressed in almost all tissues but the highest expression was recorded in unfolded young leaf (TPM 102580). Its sequence was validated by Sanger sequencing (Fig. S4B, see online supplementary material). Considering its significantly high expression in leaf, and non-specificity in terms of tissues highlight that it might be a critical gene from pepper growth and development, which should be further explored. These findings indicate that pepper plant development involves different numbers of transcripts in each stage and tissue.

We further visualized the expression trends within the tissue over time by performing PCA for each tissue separately and found different degrees of clustering (Fig. S5A–H, see online supplementary material). The PCA analyses showed that there was a marked separation of gene expression in some of the stages in individual tissues. For example, a clear separation in the bud (FL1-B) and flower stalk (FL1-A) at the bud stage, and in the stamen (FL2 and FL3) at bud and flowering stage in flowers was observed (Fig. S5A, see online supplementary material). Which is indicative of different growth mechanisms and expressions in the stalk and bud. In the case of fruit, tissues at different DAF showed lower variation with time (FA1-FA5). However, from 30 DAF to 55 DAF, the PCA plots showed variation from earlier stages, indicating changes in expression (Fig. S5B, see online supplementary material). In the case of placenta, the maximum variation was observed on PC1 between the time of tissue sampling (Fig. S5C, see online supplementary material). Similarly, variations in carpopodium, leaf, root, seed, and stem were observed (Fig. S5D–H, see online supplementary material). These results highlight that expression is influenced by the growth stage in chili pepper within the same tissue.

Next, we explored the differential alternative splicing (DAS) events between the studied tissues; as shown in Fig. S6A (see online supplementary material), 2756 loci showing DAS events were found. The highest number of DAS were between leaf vs fruit (218) followed by leaf vs seed (190), and leaf vs placenta (189). Generally, the number of DAS between vegetative and reproductive tissues was higher. The most significant locus showing DAS between carpopodium and leaf was *gene_Capana06g002873* (ubiquitin-conjugating enzyme E2–17 kDa isoform X2), and between fruit and flower was *gene_Capana06g003059* (universal stress protein A homolog 1-like) (Fig. S6B–D, see online supplementary material). E2 enzymes play a role for the attachment of ubiquitin to cellular proteins [15], whereas USP members respond to a multitude of environmental perturbations in different organisms [16]. These observations indicate that chili peppers’ transcribed regions are complex due to AS. The fact that a number of DAS between vegetative and reproductive tissues was higher indicates that they play roles in chili pepper fruit growth and development. Expression changes during fruit development.

**Figure 3:**
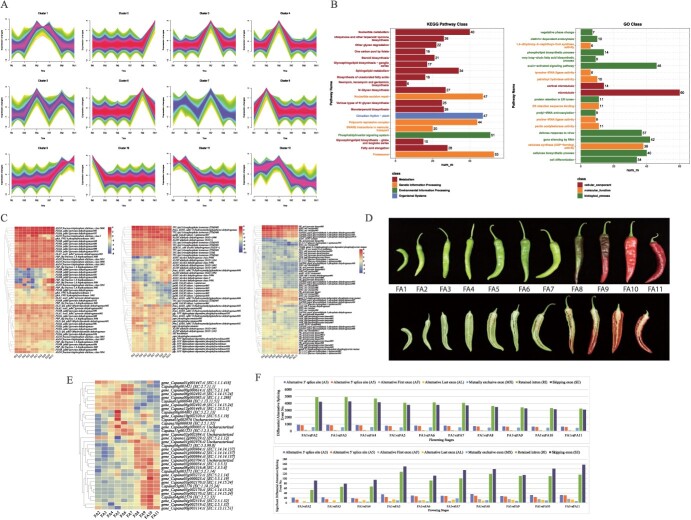
Expression changes during chili pepper fruit development. **A** Clustering analysis of transcripts expressed in fruit. **B** KEGG and GO pathway enrichment bar charts of transcripts grouped in cluster 12. Heatmaps (log2 foldchange) of the genes enriched in (**C**) glycolysis/gluconeogenesis pathway. **D** Stages of fruit development in days after flowering (DAF). **E** Heatmap (log2 foldchange) of transcripts enriched in carotenoid biosynthesis KEGG pathway. **F** Bar plots of no. of differential alternative splicing (top panel) and significantly differential alternative splicing (bottom panel).

Fruit is the edible part of chili peppers, therefore, we explored the expression changes associated with fruit development. A total of 72 911 transcripts were expressed in fruit, of which 31 777 were fruit-specific (Table S2, see online supplementary material). The expressed transcripts were grouped into 12 clusters (Fig. 3A). Specific gene clusters had the highest expressions on a specific fruiting stage (FA); for example, cluster 10 at FA1, cluster 8 and 11 at FA3, cluster 1, 4, and 5 at FA5, cluster 3 at FA7, cluster 6 at FA9, and cluster 9 and 2 at FA 11. Generally, Cluster 9 contained transcripts that showed increasing expression patterns with DAF. These genes were enriched in 96 KEGG pathways; notably protein export, porphyrin metabolism, alpha-linolenic acid, beta-alanine, D-amino acid, isoquinoline alkaloid, carotenoid, and glucosinolate biosynthesis pathways. From FA1-FA7, the pepper fruit grew in size, whereas from FA8-FA11, fruit color changed to red, while size remained almost constant. A total of 243 transcripts enriched in 60 KEGG pathways showed increasing expression from FA1-FA7. Similar to cluster 3, most transcripts of cluster 5 were enriched in metabolism-related pathways (Table S3, see online supplementary material).

Cluster 12 shows increasing expression trends till FA5 (25 DAF); that is, continuous increase in fruit size. The genes were significantly enriched in GO biological processes including cell differentiation, cellulose biosynthetic process, auxin-activated signaling pathway, vegetative phase change, etc. KEGG enrichment also showed carotenoid bio synthesis, circadian rhythm-plant, and secondary metabolism related pathways (Fig. 3B). Considering the fruit growth, the expression changes related to cell differentiation and cellulose biosynthesis were explored. Of the 65 cellulose synthesis-related transcripts (cellulose synthase A, protein COBRA-like, and endoglucanase 25-like), two transcripts showed nearly consistent expression from 5 to 30 DAF, after which their expression decreased. These expressions highlight that during fruit growth, cellulose synthesis plays a significant role. This was also consistent with cell differentiation and auxin-related (signaling, transport, homeostasis, and intracellular transport) transcripts. However, a limited number of circadian rhythm-related transcripts—phototropin-1 (*Capana11g000681*) and protein LNK4-like (*gene_Capana08g001155.t2*)—showed peak expressions only in FA5, whereas only protein ELF4-like 3-like (*gene_Capana02g002596.t1*) showed higher expressions till 10 DAF, which then continued to decrease till 50 DAF (Table S4, see online supplementary material).

Seven of the pathways where cluster 10 and 11 transcripts were enriched were related to carbohydrate metabolism signifying large-scale changes during fruit development. Of the 10 transcripts, MIOX, inositol oxygenase (*gene_Capana09g000201*), APX, L-ascorbate peroxidase (*gene_Capana06g002525*), and malate dehydrogenase (oxaloacetate-decarboxylating) (NADP+) (*gene_Capana10g001751*) had significant reduction in expression from 5 to 55 DAF (Fig. 3C; Table S4, see online supplementary material). MIOX converts myo-inositol to D-glucuronate, which is then directed towards amino sugar and nucleotide sugar metabolism and pentose and glucuronate interconversions [17], whereas malate dehydrogenase converts malate to pyruvate during carbon fixation [18]. The highest expressed transcripts in glycolysis/gluconeogenesis were enolase (*Capana09g002348*) and pyruvate kinase (*novel135526*). The pyruvate kinase expression decreased from 10 to 25 DAF, increased slightly on 35 DAF and then again decreased, whereas that of eno’enolase increased from 5 to 20 DAF, and then decreased with DAF. Two transcripts (*gene_Capana00g002259.t2* (IDH3, isocitrate dehydrogenase (NAD+)) and *Capana02g003587* (succinate dehydrogenase (ubiquinone) iron–sulfur subunit)) were noticeably highest, where IDH3 expression decreased slightly on 25DAF and then increased on final days (50 and 55 DAF). Similarly, succinate dehydrogenase increased with time till 25DAF and then increased on final days. This suggests that sugar metabolism and related pathways drive large-scale changes during chili fruit development similar to tomato [19], apple [20], and other plants [21]. An increasing expression trend of the transcripts enriched in the citrate cycle (Fig. S7; Table S2, see online supplementary material) was observed. This is consistent with the known role of citrate in fruit ripening [22]. Noticeably, *Capana05g002487* (20S proteasome subunit beta 3), *Capana12g000207* (RP-L18, MRPL18, large subunit ribosomal protein L18, rplR), and *gene_Capana03g001736.t1* (NADH dehydrogenase (ubiquinone) Fe-S protein 6, NDUFS6) had the highest TPM values. NDUFS6 is an integral part of mitochondrial electron transport and has electron transfer activity. The highest TPMs of these genes/transcripts indicates their (and, therefore, the pathways’) roles in fruit development. A large number of transcripts (2607) whose expression peaked at FA7 and then decreased were enriched in fructose and mannose metabolism, pentose phosphate pathway, glycine, serine, and threonine metabolism, phenylalanine, tyrosine and tryptophan metabolism, etc.

Based on visible change in fruit color (Fig. 3D), and the fact that carotenoid are dominant pigments in pepper fruits [23], expression changes in related genes/transcripts were studied. A total of 221 transcripts enriched in carotenoid biosynthesis were expressed in fruit throughout development. Notably, several genes had multiple transcripts expressed at different DAF. For example, abscisic acid 8′-hydroxylase (*CYP707A*) had seven transcripts of which transcript *gene_Capana01g000984.t3*’s started expressing (TPM ≥1), expressed at 25, 40, 45, 50, and 55 DAF, whereas transcripts t1 and t2 only expressed at 40 DAF. Other transcripts were either not expressed at all or TPM was <1. Of the 221 transcripts, 35 had TPM ≥1 in one or more DAF, hence their expressions were studied. Generally, most of the transcripts had increased expression from 25 DAF and reached their highest on 50 DAF, such as lycopene beta-cyclase (*gene_Capana05g000023.t1* and *gene_Capana10g002320.t1*), which is consistent with the redness of the fruit. Two beta-carotene 3-hydroxylase (*gene_Capana06g002492*) transcripts—t5 and t9—showed continued increase in expression from 5 DAF till 35 DAF, while t1, t2, and t3 started highly expressing from 40 DAF and reached maximum on 50 DAF. Almost similar trends were noted for 15-*cis*-phytoene synthase, beta-carotene isomerase (DWARF27), zeta-carotene desaturate, and violaxanthin de-epoxidase. These observations indicate the involvement of different transcripts at different fruit development stages in color development (Fig. 3E).

As multiple transcripts were detected in most annotated genes, AS events were supposed to frequently occur during fruit development. Of the seven types of AS events, SE was the most frequent type, followed by RI, A3, A5, MX, AF, and AL between FA1 and other FAs (FA2–11) (Fig. 3F). SE results in complete skipping of one or more exons and is the most common type of AS in eukaryotes [24]. Noticeably, the proportion of the significantly different AS events was approximately the same for the different DAF comparisons. Across the comparisons, the highest no. of DAS events was enriched in ribosome, followed by carbon metabolism, protein processing in endoplasmic reticulum, and spliceosome KEGG pathways (Fig. S8, see online supplementary material).

**Figure 4:**
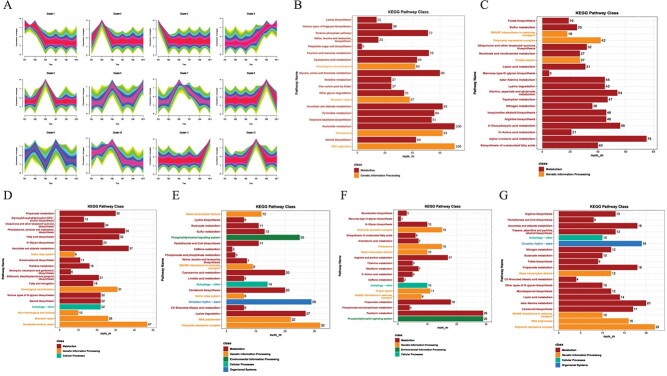
Expression changes in chili pepper placenta development. **A** Cluster analysis of the transcripts expressed in placenta. Barplots of KEGG pathway enrichment analysis of the transcripts from (**B**) cluster 1, (**C**) cluster 4, (**D**) cluster 2, (**E**) cluster 5, and (**F**) cluster 6, and (**G**) cluster 7.

These results highlight that fruit growth is accompanied by increased transcript expression in cell wall (cellulose) synthesis, cell differentiation, carbohydrate metabolism, and citrate cycle, whereas after 35 DAF, the transcripts related to pigment synthesis and accumulation and other secondary metabolite synthesis related pathways are highly expressed till maturity. Our results based on ONT also indicate that different transcript isoforms are expressed during different fruit growth and development stages. Moreover, AS events are frequently identified in pepper fruits by long-read RNA sequencing.

#### Expression changes during placenta development

Placenta development is important from both the size as well as capsaicin accumulation. Therefore, the genes that show continued increase with fruit development or vice versa can highlight key mechanisms involved. Of the 83 011 expressed transcripts, 34 556 were placenta-specific (Table S5a, see online supplementary material). Of the 12 clusters, clusters 1 and 4 showed continued decreasing and increasing expression trends, respectively (Fig. 4). Cluster 4 transcripts were highly significantly enriched in biosynthesis of unsaturated fatty acids, alpha-linolenic acid, D-amino acid, 2-oxocarboxylic acid, nitrogen, and tryptophan metabolism. This signifies higher metabolic activity during placenta development; hence the expression changes in the associated genes were explored. Noticeably, most of genes enriched in unsaturated fatty acid biosynthesis were acetyl-CoA acyltransferase 1 transcripts. Their expression mainly increased from 15 till 55 DAF. This gene can initiate a key initiation step in mevalonate pathway for terpenoid biosynthesis [25]. These transcripts were also enriched in alpha-linolenic acid metabolism along with acyl-CoA oxidase (six transcripts), alcohol dehydrogenase class-P (26 transcripts), enoyl-CoA hydratase, OPC-8:0 ligase 1, and phospholipase A1. Together with the cluster 1 transcripts enriched in steroid, proteasome, nucleotide metabolism, and terpenoid backbone biosynthesis, the data shows both primary and secondary metabolism-related expression increases with the fruit and placenta development (Table S5b, see online supplementary material).

Cluster 2 transcripts’ expression increased from 5 to 15 DAF and then decreased, whereas cluster 10 transcripts showed continuous increase from 5 to 25 DAF after which their expression decreased till 55 DAF. Similarly, clusters 5, 6, and 7 peaked at 25 DAF. Remarkably, the top-5 significantly pathways to which cluster 2 transcripts were enriched included nucleotide excision repair, autophagy, mismatch repair, non-homologous end-joining, and steroid biosynthesis. Cluster 10 genes showed continuous increase from 5 to 25 DAF, after which their expression decreased till 55 DAF (Fig. S9, see online supplementary material). Earlier studies have shown that capsaicin accumulation is higher in late maturity of placenta [26]. Clusters 12, 8, and 11 showed peak expressions at 35, 45, and 55 days, respectively, indicating their possible roles in placenta maturity and capsaicin accumulation.

Considering that several transcripts were detected for different genes enriched in key pathways (Table S5b, see online supplementary material), AS events were supposed to frequently occur during placenta development. Similar to the AS events observed in fruit development, SE was the most frequent type, as observed in the case of fruit, followed by RI, A3, A5, MX, AF, and AL between FB1 and other FB stages (FB2–11) (Fig. S9A, see online supplementary material). Noticeably, the proportion of the significantly different AS events was approximately the same for the different DAF comparisons (Fig. S9B, see online supplementary material).

**Figure 5:**
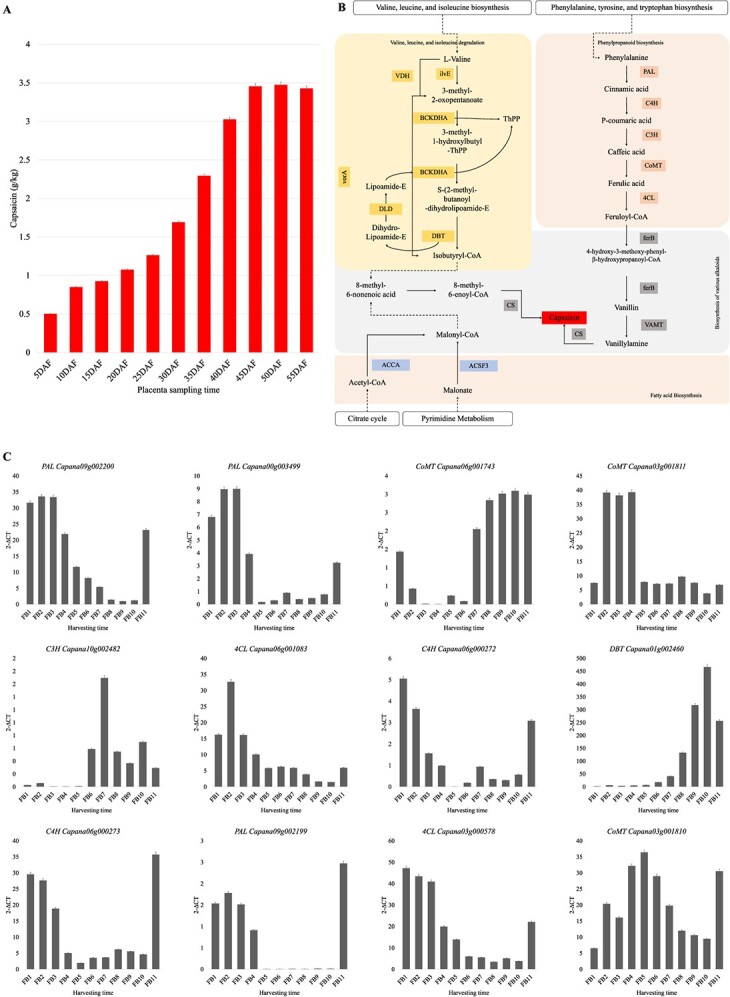
Capsaicin biosynthesis in chili pepper fruit (placenta). **A** Capsaicin content in placenta. **B** Capsaicin biosynthesis pathway (log2 foldchange values are given in Table S6, see online supplementary material). **C** Validation of the expression, of genes involved in capsaicin biosynthesis in placenta of chili pepper, by qRT-PCR. Gene/transcript names are preceded by abbreviation of annotations. 4CL, 4-coumarate—CoA ligase; ACCA, acetyl-CoA carboxylase carboxyl transferase; ACSF3, malonyl-CoA/methylmalonyl-CoA synthetase; BCKDHA, 2-oxoisovalerate dehydrogenase; C3H, C3’H`5-O-(4-coumaroyl)-D-quinate 3′-monooxygenase; C4H, trans-cinnamate 4-monooxygenase; CoMT, caffeic acid 3-O-methyltransferase; CS, capsaicin synthase; DBT, 2-oxoisovalerate dehydrogenase; DLD, dihydrolipoamide dehydrogenase; ferB, feruloyl-CoA hydratase/lyase; ilvE, branched-chain amino acid aminotransferase; PAL, phenylalanine ammonia-lyase; VAMT, vanillin aminotransferase; VDH, valine dehydrogenase (NAD+); vorA, 2-oxoisovalerate ferredoxin oxidoreductase.

Capsaicin is a pungent compound present in chili pepper fruits. Pungency (hotness) of chili peppers is associated with capsaicin content [9]. Because capsaicin biosynthesis and accumulation in different cultivars (genotypes) differs, the ONT-based transcript data during placenta development was explored to understand its biosynthesis in *C. annuum* L. var. 8214. To this regard, the capsaicin content during the placenta development was determined. Capsaicin content was minimum at FB1 stage (0.5 g/kg) and reached maximum at FB10 (3.47 g/kg), and then slightly decreased in FB11 (3.42 g/kg) (Fig. 5A). Generally, it showed a consistent increasing trend with the fruit development. Next, expression changes of the pathways that converge on capsaicin biosynthesis—various alkaloids (KO00996) including phenylpropanoid biosynthesis, and valine, leucine, and isoleucine degradation—were studied. Overall, 435 transcripts annotated as 108 KO terms were enriched in these pathways. In the case of valine, leucine, and isoleucine biosynthesis pathways, the first step is the conversion of L-valine to 3-methyl-2-oxobutanoate by the action of ilvE (branched-chain amino acid aminotransferase). Fourteen ilvE transcripts were detected in placenta; 13 of which belonged to the same gene (*gene_Capana02g002928*). Notably, two transcripts—t3 and t12—showed upregulation with growth period. 3-methyl-2-oxobutanoate is converted into S-(2-methyl-propanoyl)-dihydrolipoamide-E in two steps by the action of 2-oxoisovalerate dehydrogenase E1 (BCKDHA). Of the 18 BCKDHA transcripts, *gene_Capana12g002920.t9, gene_Capana06g001783.t3,* and *Capana04g001513* showed increasing expression trends with time till 45–50 DAF (Fig. 5B). Isobutyryl-CoA is also converted back to ThPP by the action of dihydrolipoyl dehydrogenase (DLD), which is then again converted to S-(2-methyl-propanoyl)-dihydrolipoamide-E in two steps by the action of BCKDHA. Two of the 17 DLDs transcripts (*gene_Capana08g001599.t8* and *t9*) showed upregulation with growth period. In the next step, isobutyryl-CoA is synthesized by the action of the 2-oxoisovalerate dehydrogenase E2 component (DBT). Interestingly, all the transcripts showed significant upregulation from 40 DAF. One DBT transcript (*gene_Capana01g002460.t10*) showed consistently increasing expression with growth period (Fig. 5B; Table S6, see online supplementary material). Considering their presence upstream of the capsaicin biosynthesis, these expression changes highlight that valine degradation-related genes play an active role.

Similarly, of the 11 phenylalanine ammonia lyase (PAL) and 31 4-coumarate-CoA lyase (4CL) transcripts, five 4CLs (*gene_Capana07g000029.t1*, *gene_Capana11g000130.t2*, *t5*, *t7*, and *t9*) and three PAL (*Capana03g003491*, *t1*, and *t2*) transcripts had increasing expression trends with DAF. Whereas, one capsaicin synthase transcript (*gene_Capana08g00145.t1*) showed upregulation from 10 to 25 DAF. Indicating, early induction of this transcript plays role in capsaicin biosynthesis. Whereas *gene_Capana01g002936.t1* and *t2* transcripts showed almost consistent TPM values. Caffeic acid 3-O-methyltransferase (*Capana06g001743*) transcripts, had increasing expression pattern. The expression trends of several of the genes involved in these pathways were validated by qRT-PCR (Fig. 5C). The genes that contribute malonyl-CoA from citrate cycle (acetyl-CoA carboxylase carboxyl transferase, ACCA) and pyrimidine metabolism (malonyl-CoA/methylmalonyl-CoA synthetase, ACSF3) had also shown increasing expression trends with DAF (Table S6, see online supplementary material). Malonyl-CoA is converted to 8-methylnon-6-enoyl-CoA before being converted to capsaicin. These observations indicate that capsaicin biosynthesis in *C. annuum* var. 8214, derives resources mostly from valine, leucine, and isoleucine degradation as well as citrate cycle and/or pyrimidine metabolism. This is also consistent with the increasing concentration of genes/transcripts enriched in citrate cycle (Fig. 4D).

### An online database to facilitate the exploitation of the large transcriptome data

**Figure 6:**
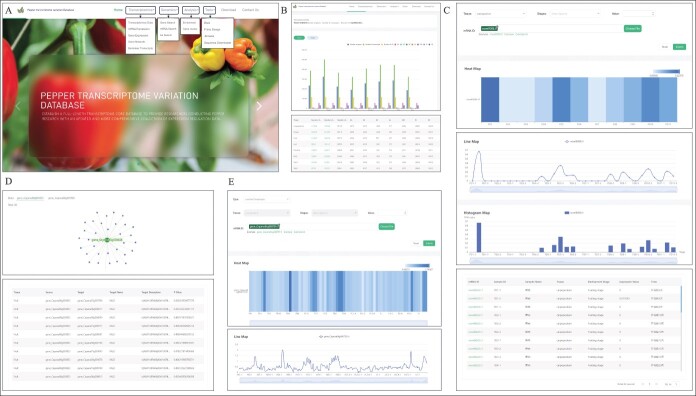
Transcriptomics functions implemented in Transcriptome data of PFTVD 1.0. **A** The home page of the PFTVD 1.0. The banner menus show functions included in each tab. Most analyses in ‘Transcriptomics’ will provide the output shown in panels **B**–**E**: **B** transcriptome data; **C** mRNA expression; **D** gene network; and **E** biomarker transcripts.

To provide seamless access to the full spectrum of transcriptome data, the Pepper Full-Length Transcriptome Variation Database (PFTVD 1.0) (http://pepper-database.cn/) has implemented powerful search capabilities and a genome browser. Overall, the PFTVD 1.0 provides functionalities covering transcriptomics, genomics, and analysis (Fig. 6A).

#### Transcriptomics and genomics

Pepper transcript expression and variation data can be accessed from the ‘Transcriptomics’ tab, which includes mRNA expression, gene expression, gene network, and biomarker transcripts. The transcriptome data can be searched and visualized by tissue or stage (Fig. 6B). The search bar requires an mRNA ID (e.g. *novel9285.t1*) or gene IDs. The output is presented as a heat map, line map, histogram, and table (Fig. 6C). A gene network based on Pearson’s correlation can be visualized (interactive network figure and table) in each tissue in the ‘Transcriptomics tab > Gene network’ (Fig. 6D). The biomarker function in the Transcriptomics tab allows users to select the type of transcript (Type > common transcripts / specific transcripts / biomarker transcripts). The user can select tissue and stage. The query is in the form of mRNA ID; multiple queries can be run simultaneously by uploading a .txt file using ‘Choose File’ in the ‘mRNA ID’ search bar. The output is provided in the form of the three types of graphs and a table (Fig. 6E). For all functions included in ‘Transcriptomics’, data can be visualized by hovering the mouse over the graphs/charts.

The Genomics tab contains five types of searches: gene, mRNA, AS, TF, and PPI. The gene or mRNA search can be performed by typing the query as a gene name, e.g. *gene_Capana00g000002*, and the search results will appear as a table. Selecting the gene from the table will produce output as ‘Overview’, ‘Structure’, ‘Sequence’, ‘Annotation’, and ‘Expression’. Within ‘Overview’, three tools are implemented: BLAST, PRIMER, and JBROWSE. The search for AS of genes selected for each tissue and stage produces the output in tabular form as for the gene/mRNA search. In addition, the types of AS within a tissue can be viewed as a pie chart. Alternative splicing events for individual genes are visualized as a structure of transcripts showing transcript, exon, and CDS (Fig. S10, see online supplementary material).

#### Analysis and tools

**Figure 7:**
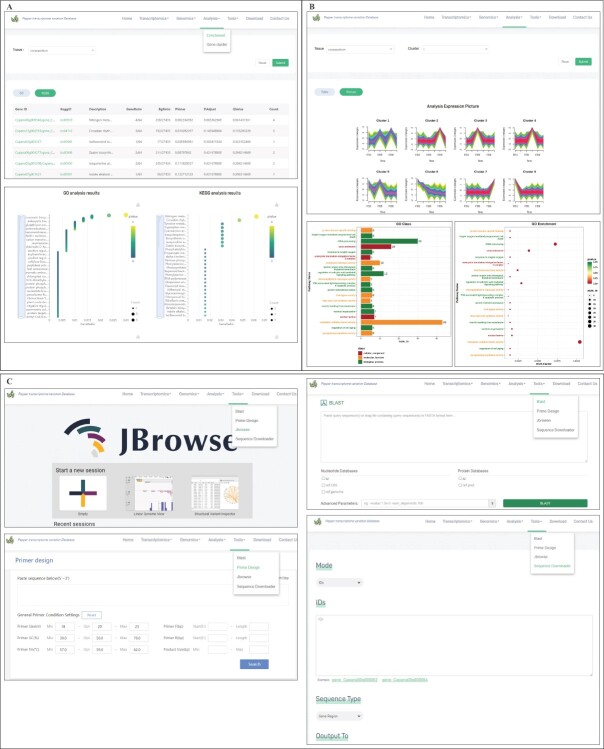
Analysis functions implemented in Transcriptome data of PFTVD 1.0. **A** Analysis page with the enrichment function selected. The KEGG enrichment is selected and the details of the genes expressed in carpopodium tissue is presented as table. GO and KEGG enrichment analyses of the genes is presented as figures, which can be directly downloaded. **B** Cluster 1 of expressed genes in carpopodium when gene cluster option is chosen from analysis menu. Top of the panel B is showing cluster analysis as graphs and the bottom is showing the GO enrichment of the selected gene cluster. **C** Tools implemented in PFTVD 1.0.

To facilitate genomic analyses, we have implemented enrichment and gene cluster analyses in the ‘Analysis’ tab, and BLAST, primer design, Jbrowse, and sequence downloader in the ‘Tools’ tab. In analysis > enrichment, users are able to choose the tissue type. In each tissue type a list of genes is displayed together with GO and KEGG analyses. Enrichment analysis results are displayed as interactive scatter plots and bar charts, and have direct links for downloading (Fig. 7A). The analysis > gene cluster enables users to choose tissue type and within each tissue, individual clusters can be selected. An output of the genes in each cluster is then produced as a table or picture together with GO and KEGG analysis (and enrichment) results (Fig. 7B–C).

## Discussion

Here, we report full-spectrum spatiotemporal transcriptome sequence of eight chili pepper tissues on five plant growth stages. Existing databases on chili pepper gene expression are limited to expressed sequence tags [7] and second-generation sequencing [8]. Our comprehensive work consisting of 488 054 full-length transcripts provides value-added information and will therefore be useful in a user-friendly way. The detection of a higher number of lncRNAs and non-coding RNAs indicates their significant role in chili pepper development. These RNAs have been implicated in the regulation of plant development, particularly ovule, seed, and fruit development [27]. Apart from these, the detection of higher numbers of protein-coding RNAs and processed transcripts has not been achieved before. Thus, our data provide a full landscape of expressed transcripts in chili pepper at the tissue and developmental stage levels. The discovery of novel transcripts is indeed a step forward compared to the previous reports in *Capsicum frutescens* and *C. annuum* chili peppers (Fig. 2) [8, 28]. The possible explanation for this could be the sequencing depth and technology. Another possible explanation could be that the number of detectable transcripts in our study were based on a large number of samples belonging to different growth stages and tissues. Similar observations have been made in other organisms while covering the full spectrum of transcriptomes: chicken, mice [13, 29], tomato [30], and triticale [31]. ONT requires a relatively lower number of reads (7.5 million) compared to short-read and second-generation RNA seq. This feature enables the increased use of long-read sequencing to be economically more feasible [32]. Our study generated ~7.56 million reads, indicating sufficient coverage. This interpretation is also supported by the detection of 1 to ≥8 transcripts in novel genes. As in other higher organisms, especially fruit plants, our results indicate that chili peppers’ transcribed regions are complex due to AS. Their roles in evolution, diversity in traits of interest, and growth and development has been well established in several plant species such as tomato [30], other fleshy fruits [33], strawberry [34], and even in chili pepper [35]. Thus, the use of long-read sequencing in our study has significantly increased the amount of generated information on chili pepper transcriptome.

When analysing the RNA-seq data across the tissues, the effective clustering of similar tissues together, and the variation in growth stages indicate stage and tissue specificity [2]. These observations suggest variable and tissue-specific growth and development on different stages, which is consistent with the magnitude and enrichment of expressed transcripts. This is particularly relevant to the observations related to transcripts showing increasing expression trends of GO terms associated with sucrose alpha-glucosidase activity, sucrose metabolic processes, seed maturation, and carotenoid biosynthesis [36], whereas the decreasing expression trends of cluster 3 and 5 transcripts associated with phenylalanine, tyrosine, and tryptophan biosynthesis, photosynthesis: antenna proteins, glycan degradation, fructose and mannose metabolism pathways are consistent with the developmental changes in chili fruit [37]. Specific to tissues within chili pepper fruit, this is the first detailed data on the transcripts expressed throughout the placental development. The increasing and decreasing expression trends of genes enriched in metabolic pathways indicate high metabolic activity during placenta development, whereas the decreasing expression trends of genes enriched in valine, leucine and isoleucine biosynthesis, glycan degradation, and other metabolic pathways including steroid biosynthesis indicate resource mobilization towards pathways converging on pigment biosynthesis or capsaicinoids biosynthesis. Though capsaicinoids have also been detected in pericarp, the major tissue where they are biosynthesized and stored is placenta [38]. The genes enriched in valine, leucine, and isoleucine biosynthesis (ilvE, BCKDHA, DLD, and DBT), phenylpropanoid biosynthesis (PAL, C4H, C3H, CoMT, and 4CL), and fatty acid biosynthesis (ACCA and ACSF3) have shown consistent expression trends with the chili pepper fruit growth and capsaicin accumulation [39]. Moreover, expressions of the transcripts annotated as enolase*,* pyruvate kinase, glyceraldehyde 3-phosphate dehydrogenase, succinate dehydrogenase, ketol-acid reductoisomerase, 2-oxoisovalerate dehydrogenase E1, and acetolactate synthase highlight that capsaicin biosynthesis can be influenced by several key metabolism pathways. These are novel candidates for functional characterization both in terms of placental development and capsaicin biosynthesis.

Centralized and user-friendly databases provide value-added information to researchers with minimal requirements of advanced bioinformatics and analytical skills. Our previous efforts in this regard continue to provide a comprehensive omics resource [8]. Developments in sequencing technologies allow the generation of a full-spectrum of expression data at lower cost [40]. Although the number of transcriptome sequencing projects is increasing with the reduction in sequencing costs, most researchers choose to submit raw sequencing data to public repositories, such as Sequence Read Archive (https://www.ncbi.nlm.nih.gov/sra). However, accessing these raw files, and analysing the information therein requires skills and expertise. In such a scenario, our user-friendly ‘Pepper Transcriptome Variation Database’ addresses this lack of accessible transcriptome information. Having implemented functions related to transcriptomics (mRNA expression, gene expression, gene network, and biomarker transcripts) and genomics (gene, mRNA, and AS), this database provides tissue-specific and growth stage related expression analyses. The PFTVD 1.0 database provides ready to use figures such as GO and KEGG enrichment analysis, AS events, and gene clusters in individual chili pepper tissues. These features are not yet available for chili pepper on any database. The database offers options to download the queried data, searching the transcriptome using BLAST, designing of primers, and Jbrowse. In conclusion, the PFTVD 1.0 database provides a comprehensive, versatile, easy-to-use, and free online tool to investigate chili pepper growth stage and tissue-specific transcriptome data.

## Materials and methods

### Plant material, growth conditions, and tissue collection

Pepper (*C. annuum* L.) selfing line 8214, developed by the Hunan Vegetable Research Institute, China, was used as plant material. It has a fruit length, width, and flesh thickness of 19.2 cm, 3.4 cm and 0.32 cm, respectively, and has been widely used as a parent material (direct or indirect) in the development of 36 chili varieties. It is a late maturing, heat and drought tolerant, and highly compatible variety with strong hybrid vigor. Seeds were germinated and grown in a growth chamber/green house (16 h light and 8 h dark at 23°C and 16°C, respectively). Tissues were collected as reported earlier [2]. The stem, leaf, root, fruit, placenta, seed, carpopodium and flower samples (Table S7, see online supplementary material) were collected in triplicate from healthy plants on five stages: seed stage, seedling stage, flowering period, bud stage, and fruiting period. Seeds and placenta were separated from fruit as reported earlier [41]. It was ensured that the collected samples were free from any pathogen or insect infection. The collected samples were washed with distilled water to remove any dust particles or contamination present on surface. The samples were then immediately frozen in liquid nitrogen and stored at −80°C until further processing.

### RNA extraction, library construction, and sequencing

Total RNA was extracted from triplicate samples of chili tissue (76 × 3) using the ONT protocol. Briefly, the Invitrogen Super-Script IV first-strand synthesis system was used for reverse transcription of full-length mRNA followed by cDNA PCR for 14 cycles with the LongAmp tag (New England Biolabs, Ipswich, MA, USA). The PCR products were then subjected to FFPE DNA repair and end repair (New England Biolabs) procedures, followed by T4 DNA ligase (New England Biolabs) adaptor ligation. The ONT procedure was used to purify the DNA using Agencourt AMPure XP beads. The final cDNA libraries were loaded into FLO-MIN109 flow cells and run on the PromethION platform from Benagen Technology Co., Ltd (Wuhan, Hubei, China).

### RNA-seq data analyses

Raw reads with average read quality ≤7 and length ≤50 bp were filtered using NanoFilt V 2.8.0 and SeqKit v 0.12.0. Pychopper v 2.4.0 was used to identify, orient, and trim the full-length nanopore cDNA sequences in the valid sequencing data. The resulting full-length sequences were aligned to the reference genome [3] using Samtools v 1.11 [42]. Non-redundant transcript sets were then constructed using Flair v 1.6.2 (https://github.com/BrooksLabUCSC/flair). Novel genes and transcripts were identified by comparing the consensus sequences with known transcripts of the genome using gffcompare v 0.12.6 [43] The coding region sequences of the novel transcripts were predicted using TransDecoder software v 5.5.0 (https://github.com/TransDecoder/TransDecoder). Functional annotation of the novel transcripts was performed in seven databases including Nr [44], Pfam [45], Uniprot [46], KEGG [47], GO [48], KOG/COG [49], and KEGG Pathway [50]. Transcripts and genes were counted and plotted (all, known, and novel). Moreover, the number of transcripts contained in genes were also counted. Transcript density was measured and visualized as a density distribution map using Circlize package. The transcript types—coding RNAs, long non-coding RNAs (LncRNA), and other non-coding RNAs—were counted. Variable shear analysis was done to identify the alternative splicing types in studied samples followed by identifying differential variable splicing between tissues by using suppa2(DiffSplice) [51]. The LncRNAs were predicted using CNCI V 2.0 [52] and CPC2 V standalone_python3 v1.0.1 [53]. Expression was computed as Transcripts Per Kilobase Million (TPM). Tissue-specific transcripts were identified by using the criteria if a transcript with TPM > 1 was expressed in any five samples in each tissue, it was considered tissue-specific [14].

### Design and construction of the database

The Pepper Full-length Transcriptome Variation Database (PFTVD 1.0) is a web-based platform built using frameworks including the JDK Java framework, Vue (v. 3.0), and Node 14+. The platform was developed using Docker (v. 23.0.1) containers, running on nginx (v. 1.13.) web servers. Moreover, Vite, Sass, and Axios were used for developing, whereas technologies like D3, Echarts, and VxeTable were used for the implementation of online visualization of data. The basic databases and operating systems are MySQL (v.8.0) and CentOS (v. 7.9). JBrowse 2 was used to visualize the genome and gene structure. PFTVD 1.0 General similarity search tool (BLAST) and primer designing tool Primer 3 are also implemented in PFTVD 1.0. BLAST (v. 2.6.0) is a tool for comparing genomes, genes, proteins, and full-length transcriptional sequences. Primer 3 is a tool for primer design [54].

### Determination of capsaicin

The standard method GB/T 21266–2007 was used to determine the content of capsaicin. The peppers were ground using an electric grinder after they had first been dried to a constant weight. 5.0 g of pepper powder was weighed and put into a 100 mL beaker after being sifted at 0.391 nm. The sample was then filled with 25 mL of a methanol-tetrahydrofuran (1:1, V/V) mixed solvent, sealed with plastic wrap, and punctured several times with a needle. The sample was then subjected to an ultrasonic shock for 30 min at 60°C in a water bath. The filtrate was then collected, and the filter residue and filter paper were then added again, along with 25 milliliters of the methanol-tetrahydrofuran (1:1, V/V) mixed solvent. An ultrasonic oscillator was used to extract twice, for ten minutes each. Three rounds of filtering were used to collect the filtrate, which was then combined, concentrated to 10–20 mL at 70°C using a rotary evaporator, and filled to 50 mL with a mixed solvent of methanol and tetrahydrofuran (1,1, V/V), filtered through a 0.45 μm filter membrane for chromatographic analysis. Chromatographic separations were carried out at 30°C using a Zorbax SB-C18 (5 μm, 4.6 mm × 250 mm) column. The mobile phase was a solvent methanol–water solution (methanol: water, 65:35, V/V), with a flow rate of 1.0 mL/min.

### qRT-PCR analysis and sanger sequencing

The RNA extraction, genomic DNA removal, and first-strand cDNA synthesis was performed as reported earlier [55] using the Vazyme fluorescent quantitative kit (ChamQ Universal SYBR qPCR Master Mix, Jiangsu, China). Primers were designed based on selected sequences from RNA sequencing results using Primer 3 (Table S8, see online supplementary material). Quantitative-real time PCR reactions were carried out in triplicate. The volume was 20 μL as per manufacturer’s instructions. Actin gene was used as an internal control. 2-∆Ct method was used for computation of gene expression [56]. The selected genes with the higher TPM and the locus with significant AS were confirmed by Sanger sequencing (TA cloning).

## Supplementary Material

Web_Material_uhae198

## Data Availability

All the data generated in this study have been implemented in the Pepper Full-length Transcriptome Variation Database (http://pepper-database.cn/). Raw transcriptome data are submitted to NCBI SRA: PRJNA1046572 (https://www.ncbi.nlm.nih.gov/bioproject/?term=PRJNA1046572). Supplementary tables are available at https://figshare.com/account/home#/projects/187023.
