# Supplementary material for: Full-length transcriptome sequencing of pepper fruit during development and construction of a transcript variation database
Source: Hortic Res. 2024 Jul 24;11(9):uhae198. doi: 10.1093/hr/uhae198 (PMC11387007; doi:10.1093/hr/uhae198)
Supplement: Web_Material_uhae198 [file web_material_uhae198.zip › V3 Figure S1.pdf]

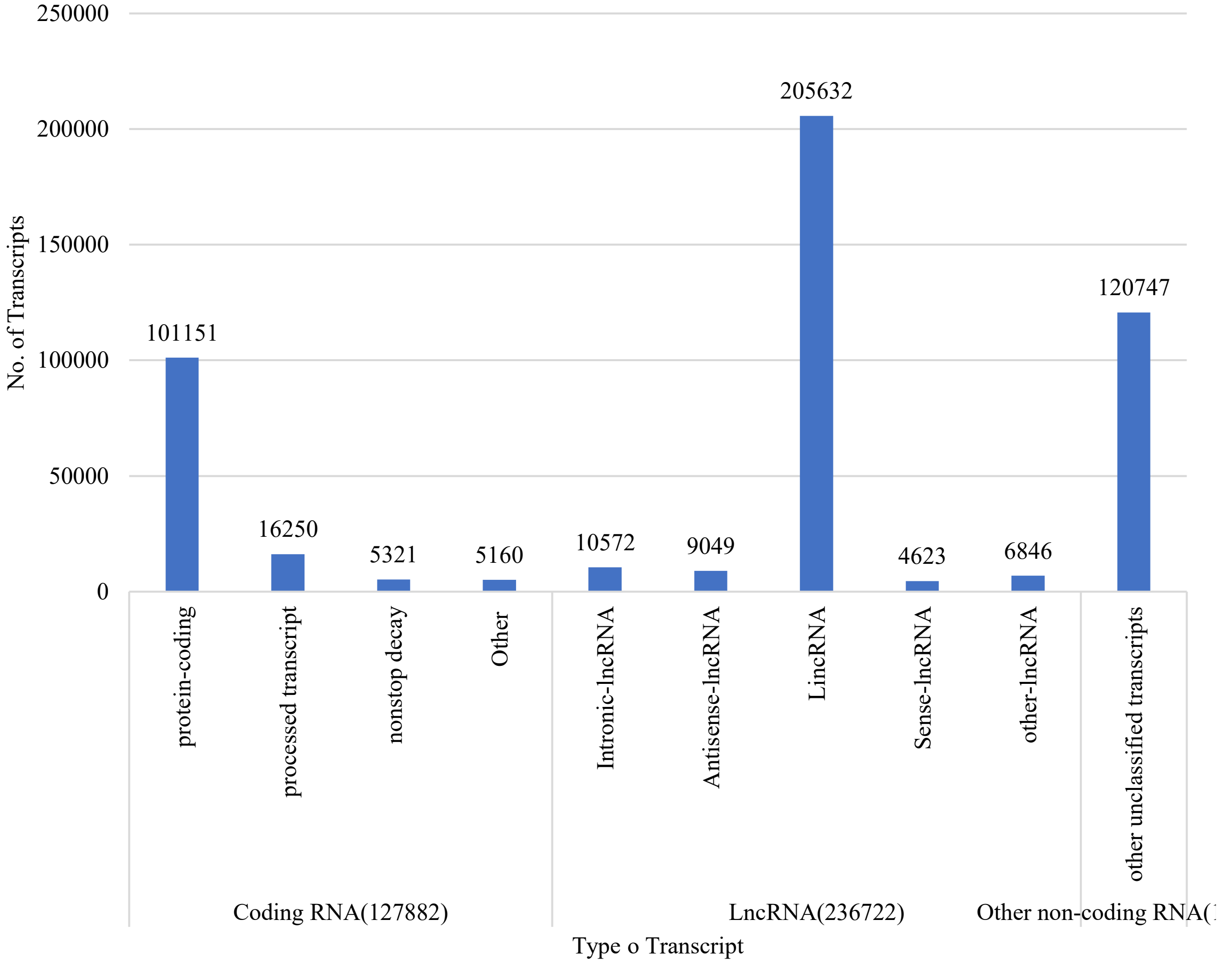

Supplementary Figure 1. Statistics of types of novel transcripts identified in chili pepper through long-read sequencing.
