# Supplementary material for: Full-length transcriptome sequencing of pepper fruit during development and construction of a transcript variation database
Source: Hortic Res. 2024 Jul 24;11(9):uhae198. doi: 10.1093/hr/uhae198 (PMC11387007; doi:10.1093/hr/uhae198)
Supplement: Web_Material_uhae198 [file web_material_uhae198.zip › V3 Figure S4.pdf]

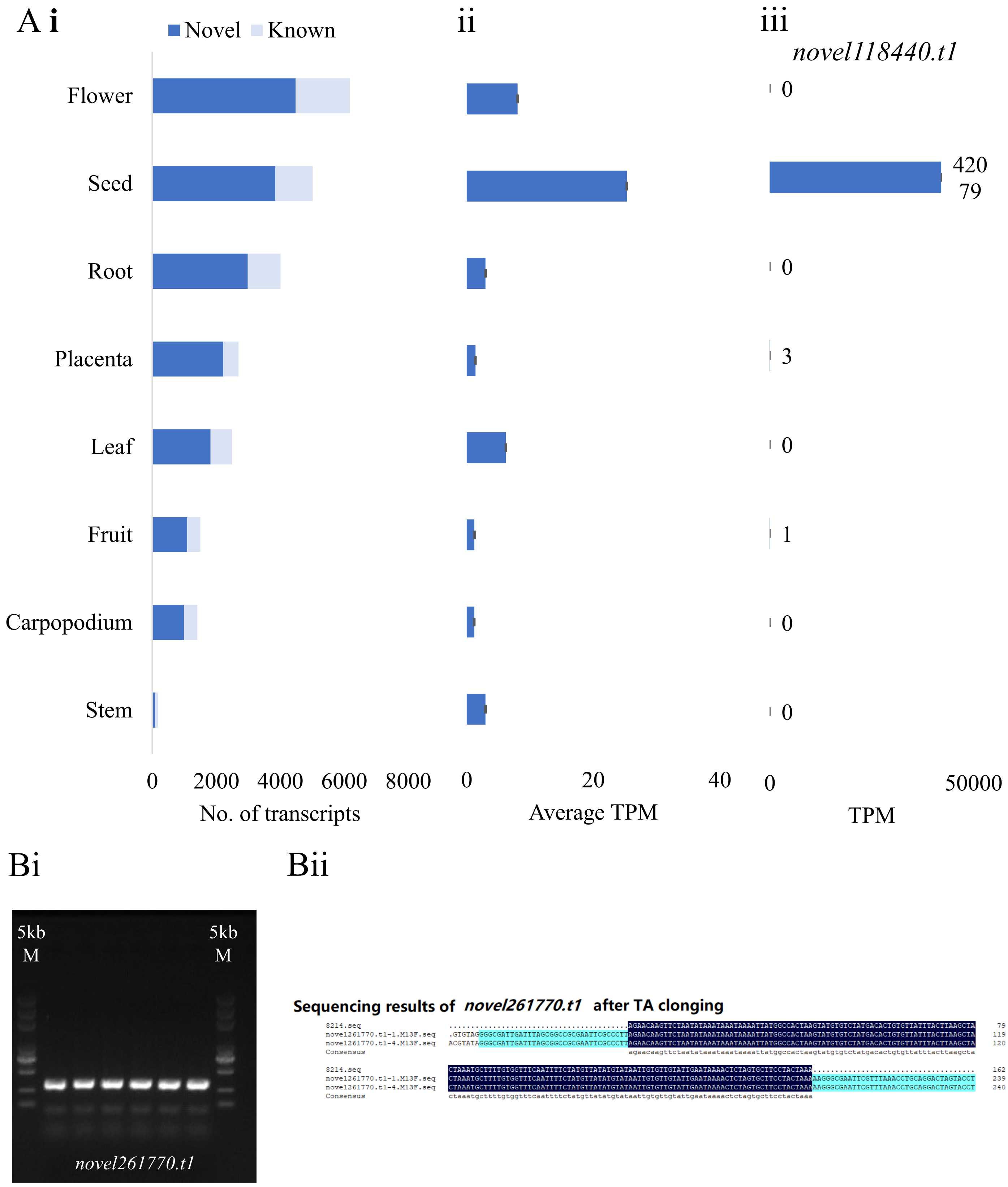
A i
Novel
Known
Flower
Seed
Root
Placenta
Leaf
Fruit
Carpopodium
Stem
0
2000
4000
6000
8000
No. of transcripts
ii
0
20
40
Average TPM
iii
novel118440.t1
0
42079
0
3
0
1
0
0
0
50000
TPM
Bi
5kb M
5kb M
novel261770.t1
Bii
Sequencing results of novel261770.t1 after TA clonging
8214.seq
novel261770.t1-1.M13F.seq
novel261770.t1-4.M13F.seq
Consensus
