# Supplementary material for: Full-length transcriptome sequencing of pepper fruit during development and construction of a transcript variation database
Source: Hortic Res. 2024 Jul 24;11(9):uhae198. doi: 10.1093/hr/uhae198 (PMC11387007; doi:10.1093/hr/uhae198)
Supplement: Web_Material_uhae198 [file web_material_uhae198.zip › V3 Figure S6.pdf]

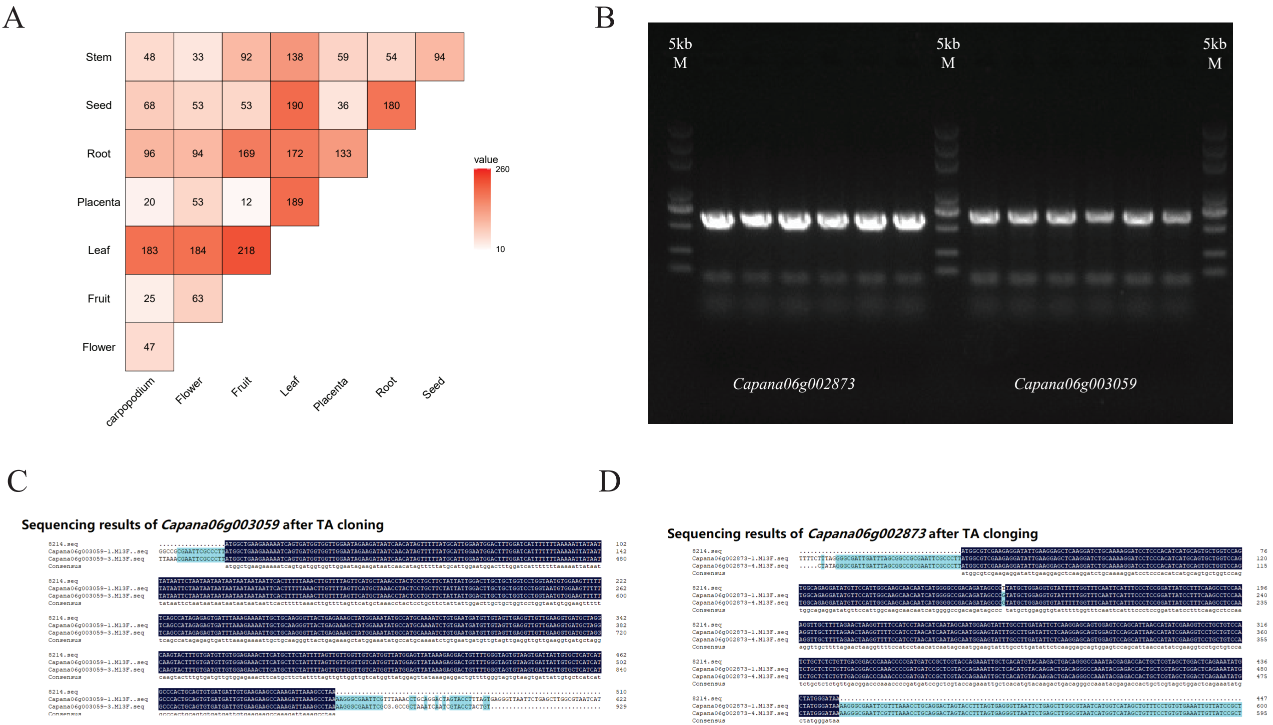
A
Stem
Seed
Root
Placenta
Leaf
Fruit
Flower
48 33 92 138 59 54 94
68 53 53 190 36 180
96 94 169 172 133
20 53 12 189
183 184 218
25 63
47
carpopodium
Flower
Fruit
Leaf
Placenta
Root
Seed
value
260
10
B
5kb M
5kb M
5kb M
Capana06g002873
Capana06g003059
C
Sequencing results of Capana06g003059 after TA cloning
D
Sequencing results of Capana06g002873 after TA clonging
