# Supplementary material for: Full-length transcriptome sequencing of pepper fruit during development and construction of a transcript variation database
Source: Hortic Res. 2024 Jul 24;11(9):uhae198. doi: 10.1093/hr/uhae198 (PMC11387007; doi:10.1093/hr/uhae198)
Supplement: Web_Material_uhae198 [file web_material_uhae198.zip › V3 Figure S8.docx]

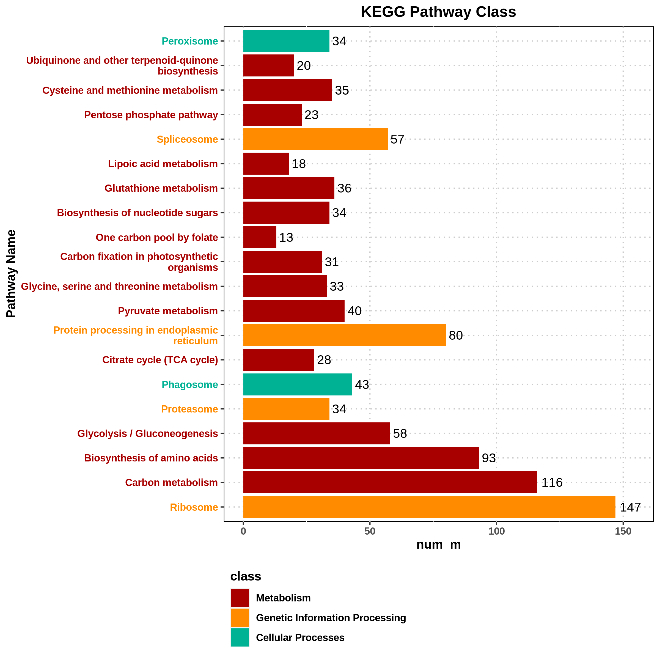

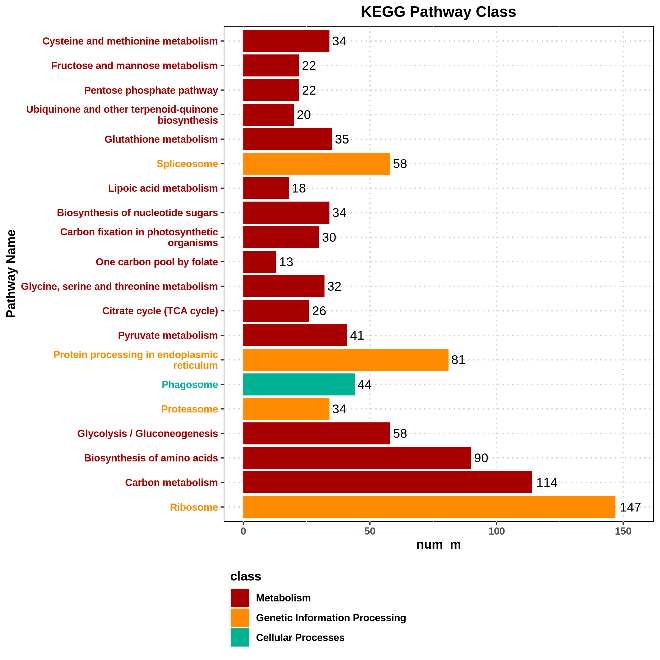


A B


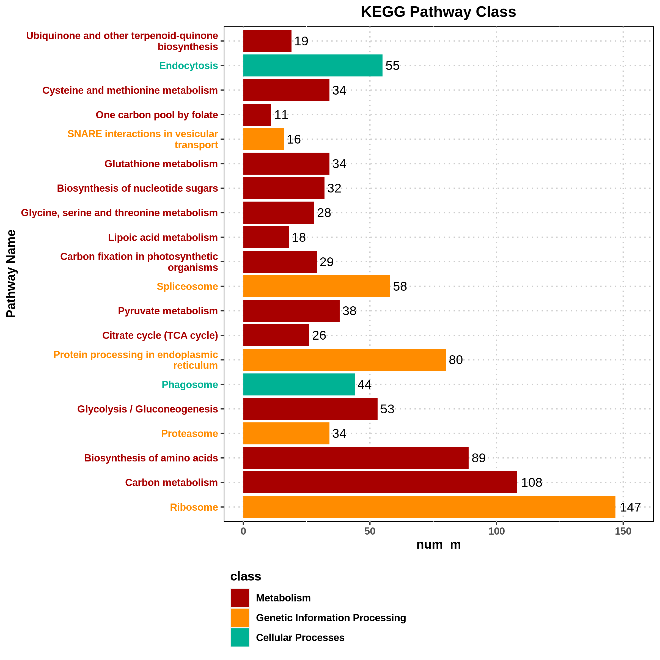

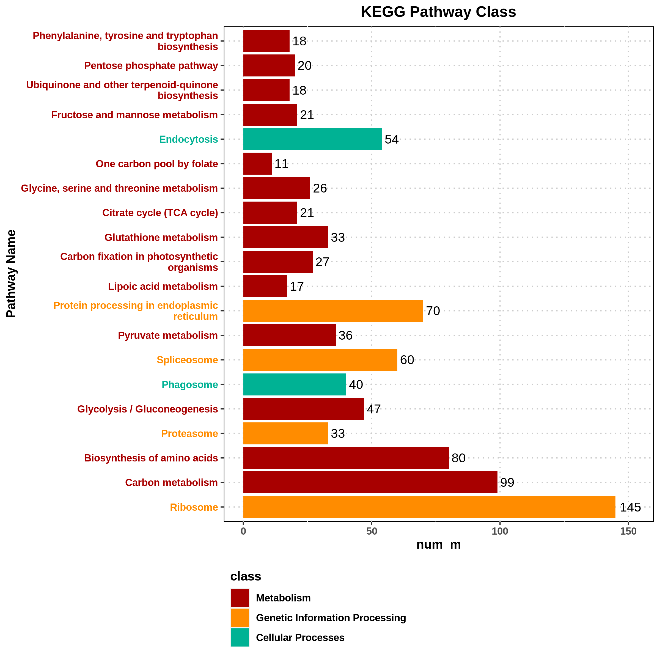


C D


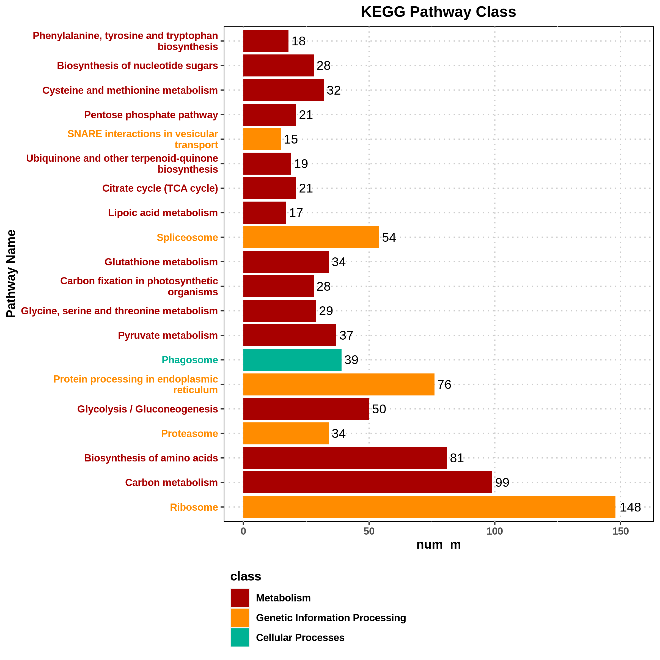

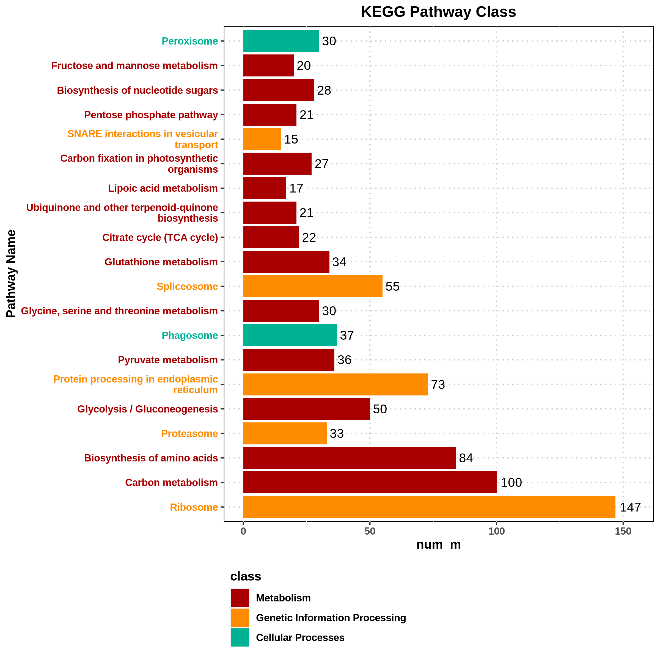


E F


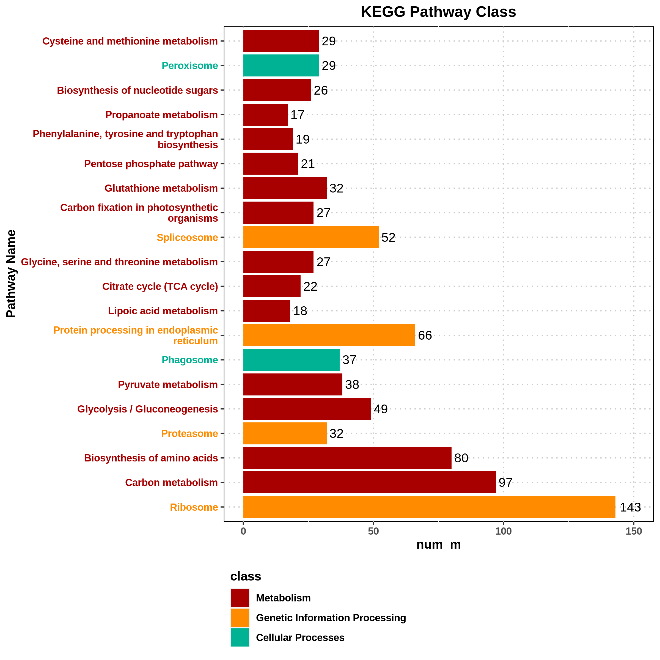

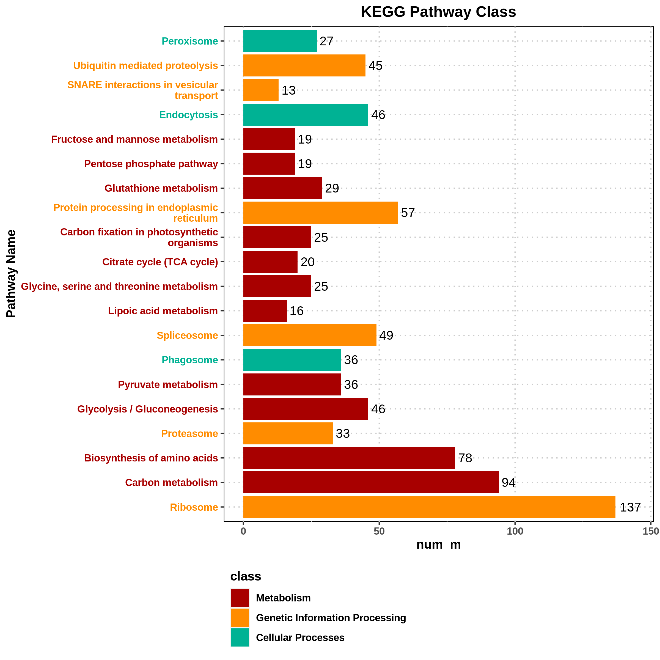


G H


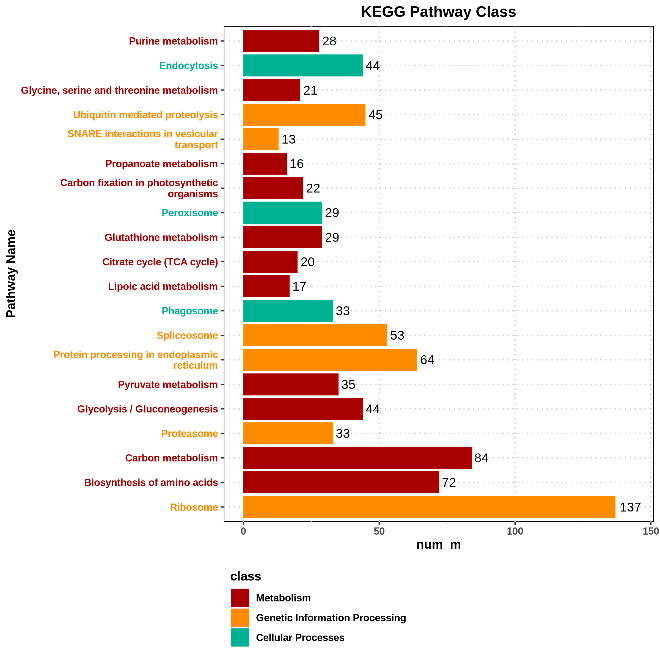

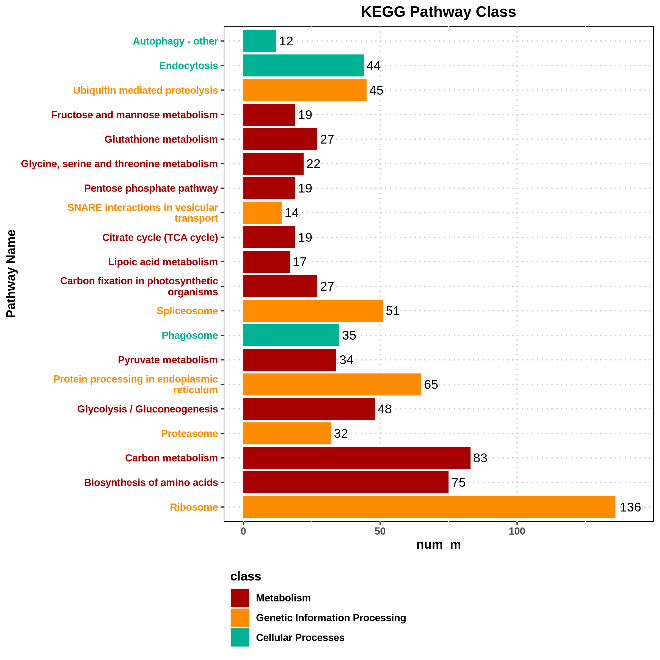


I J

**Supplementary Figure 6.** KEGG pathway enrichment of differential alternative splicing events during chili pepper fruit development. A-J; FA1vsFA2, FA1vsFA3, FA1vsFA4, FA1vsFA5, FA1vsFA6, FA1vsFA7, FA1vsFA8, FA1vsFA9, FA1vsFA10, FA1vsFA11, respectively.
