# Supplementary figures and images for: Full-length transcriptome sequencing of pepper fruit during development and construction of a transcript variation database
Source: Hortic Res. 2024 Jul 24;11(9):uhae198. doi: 10.1093/hr/uhae198 (PMC11387007; doi:10.1093/hr/uhae198)

Intersection Size

20000  
15000  
10000  
5000  
0

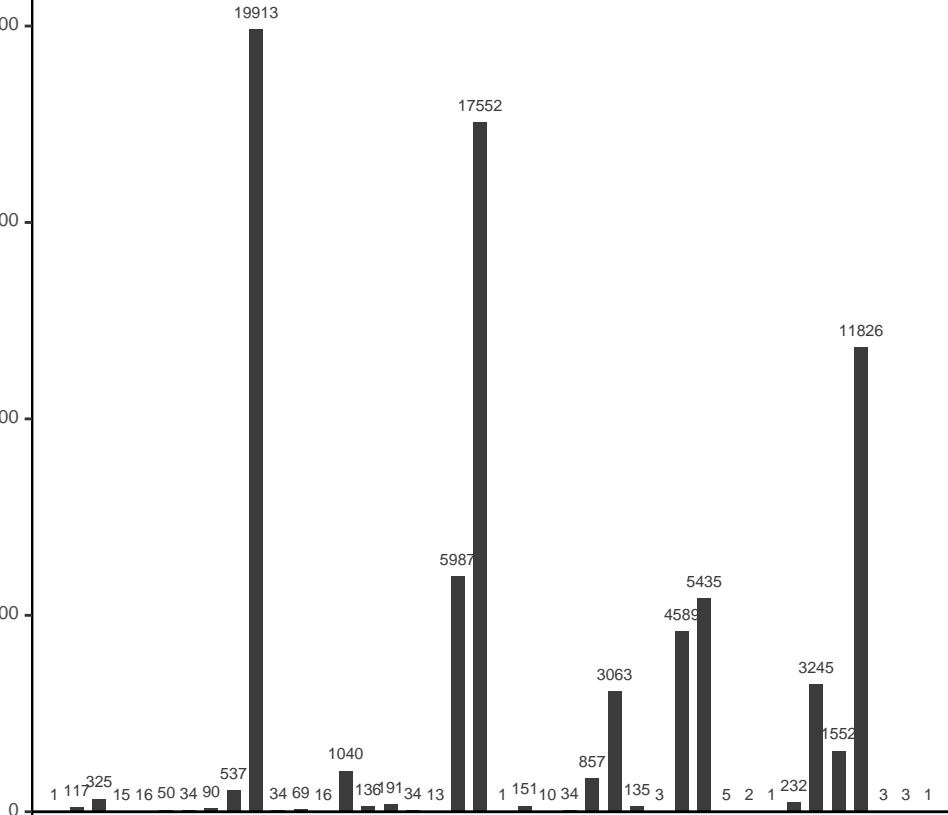

KOG  
TF  
Pathway  
Pfam  
KEGG  
GO  
Uniprot  
Nr

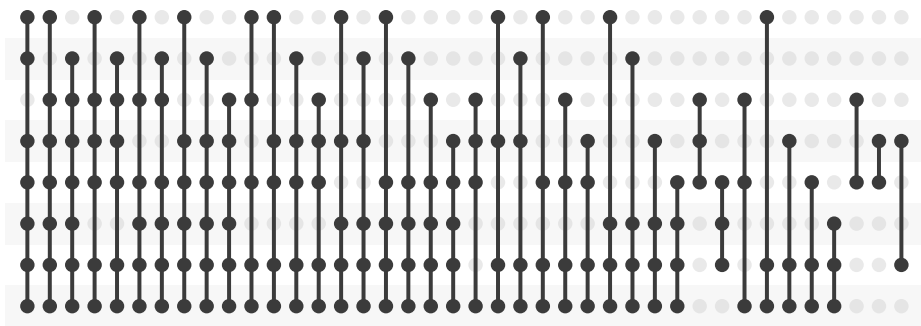

Set Size

75000  
50000  
25000  
0

Supplement: Web_Material_uhae198 [file web_material_uhae198.zip › V3 Figure S2.pdf]

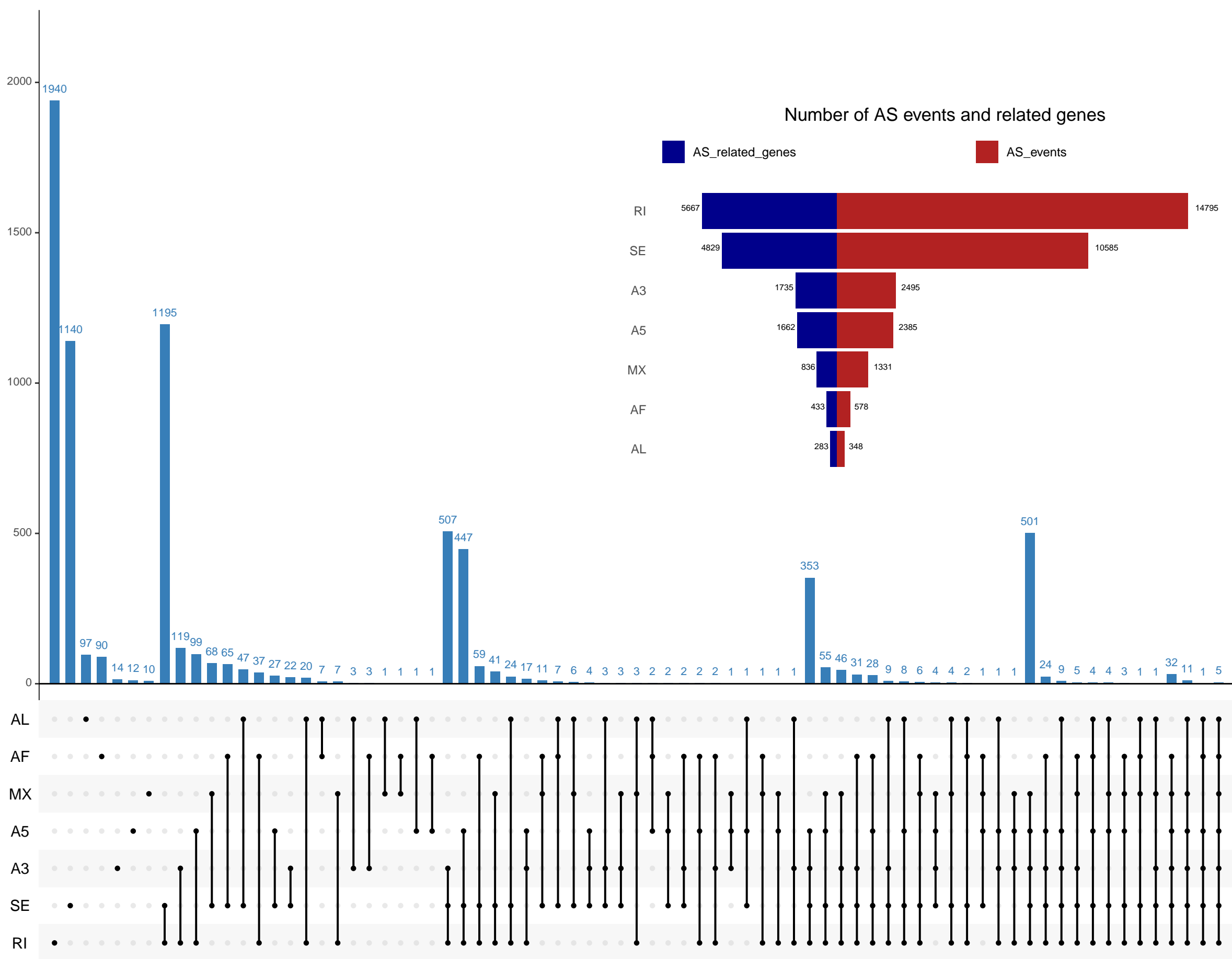

Supplement: Web_Material_uhae198 [file web_material_uhae198.zip › V3 Figure S3A.pdf]

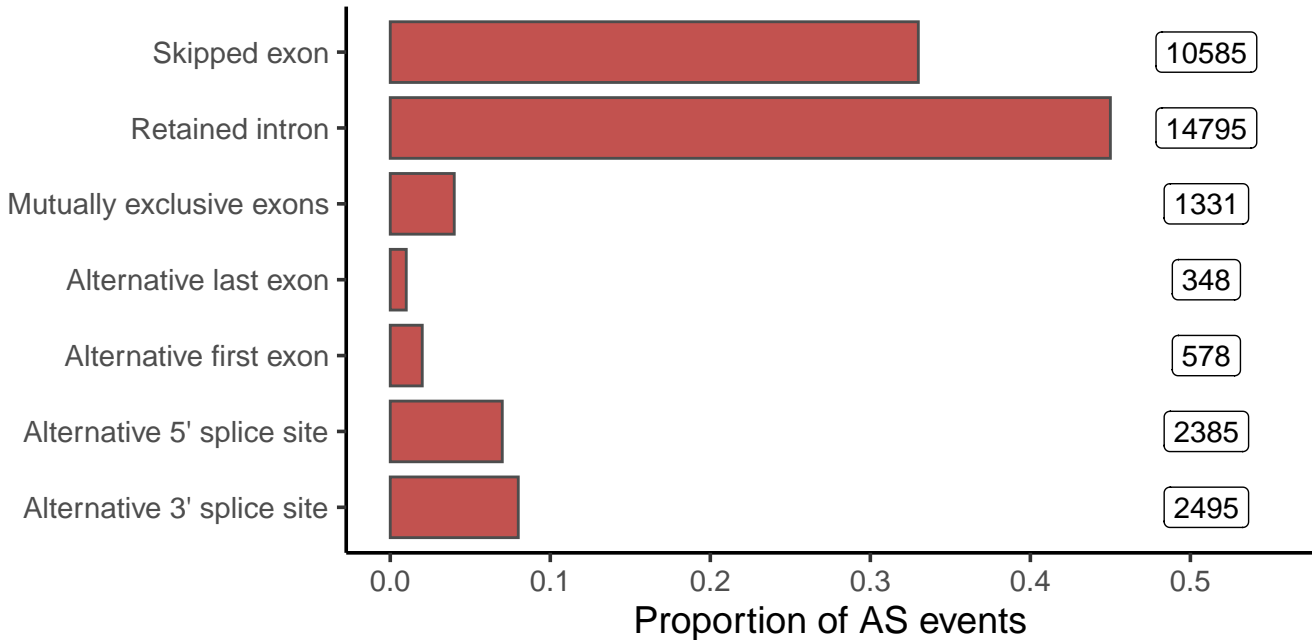

Supplement: Web_Material_uhae198 [file web_material_uhae198.zip › V3 Figure S3B.pdf]

A

2D PCA Plot

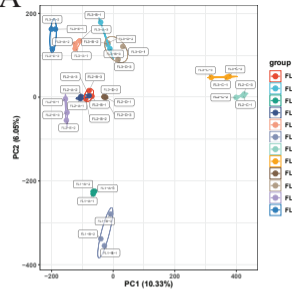

B

2D PCA Plot

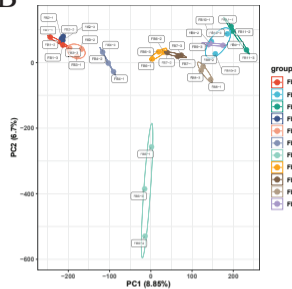

C

2D PCA Plot

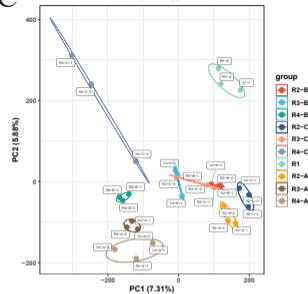

D

2D PCA Plot

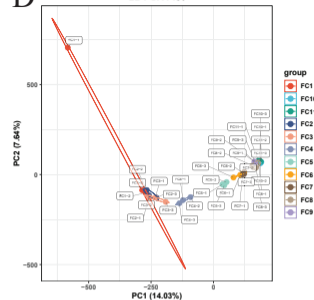

E

2D PCA Plot

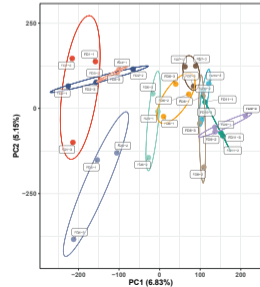

F

2D PCA Plot

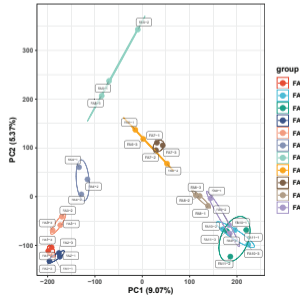

G

2D PCA Plot

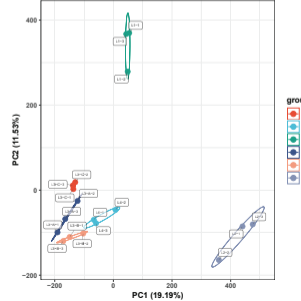

H

2D PCA Plot

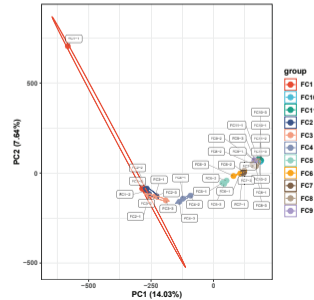

Supplement: Web_Material_uhae198 [file web_material_uhae198.zip › V3 Figure S5.pdf]

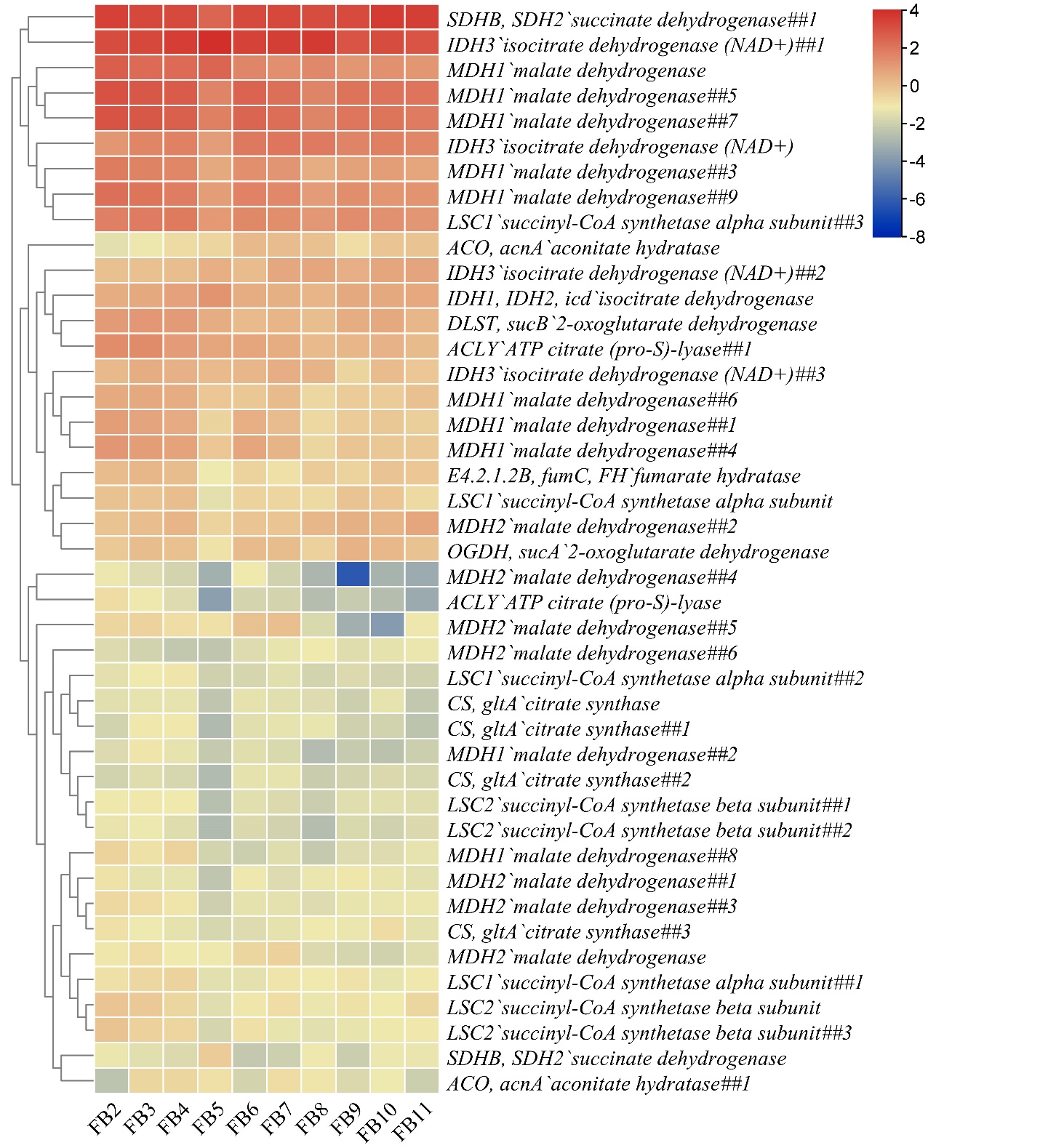


**Supplementary Figure 5.** Heatmap (log2 foldchange) of transcripts enriched in citrate cycle.

Supplement: Web_Material_uhae198 [file web_material_uhae198.zip › V3 Figure S7.docx]

## Slide 1
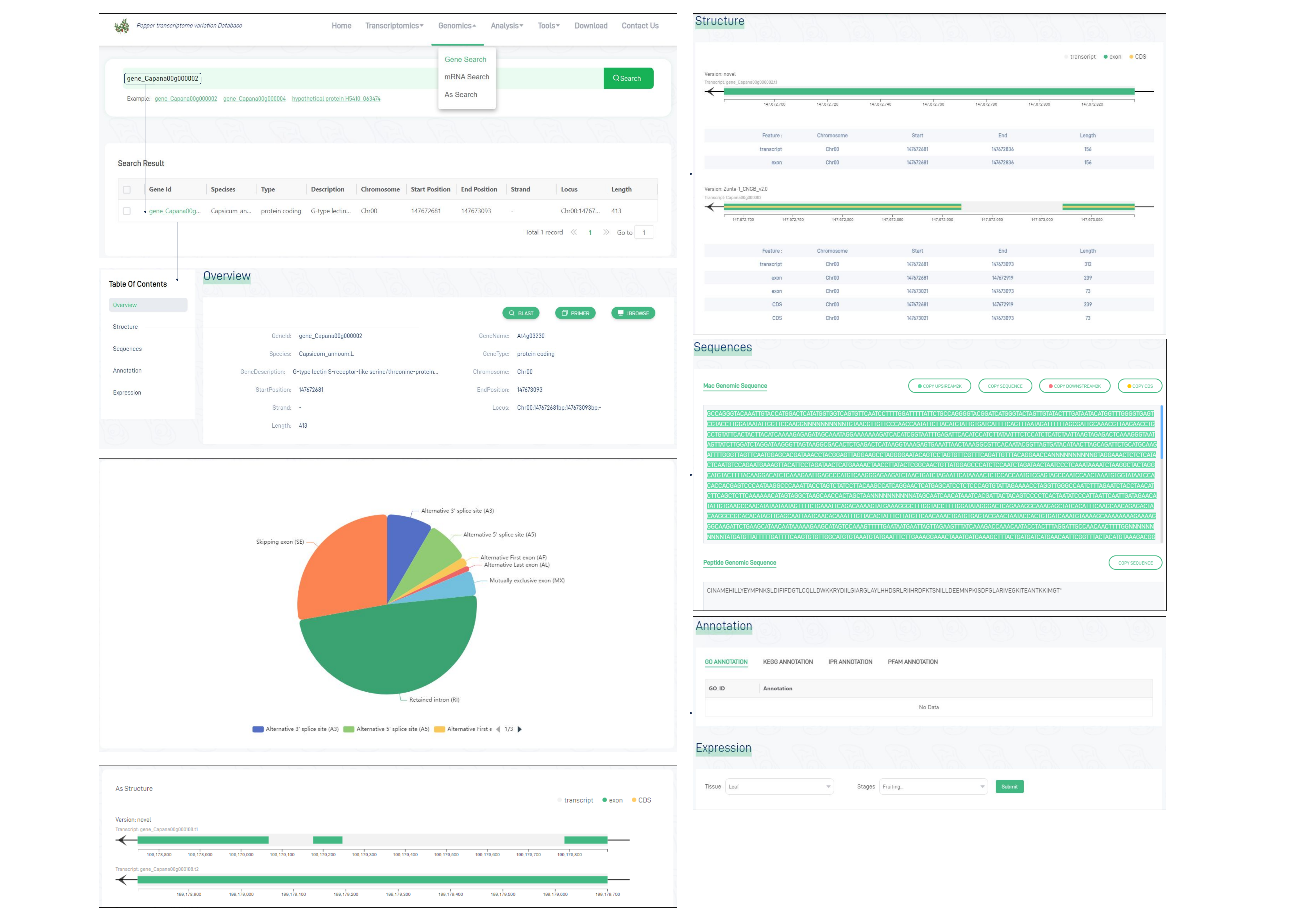

Supplement: Web_Material_uhae198 [file web_material_uhae198.zip › V3 Figure S10.pptx]
